# Associations between infant feeding and the size, tempo and velocity of infant weight gain: SITAR analysis of the Gemini twin birth cohort

**DOI:** 10.1038/ijo.2014.61

**Published:** 2014-05-06

**Authors:** L Johnson, C H M van Jaarsveld, C H Llewellyn, T J Cole, J Wardle

**Affiliations:** 1 Centre for Exercise, Nutrition and Health Sciences, School of Policy Studies, University of Bristol, Bristol, UK; 2 Health Behaviour Research Centre, Department of Epidemiology and Public Health, University College London, London, UK; 3 Department of Primary Care and Public Health Sciences, King’s College London, London, UK; 4 Population, Policy and Practice Programme, Institute of Child Health, University College London, London, UK

**Keywords:** growth, infancy, weaning, breastfeeding, SITAR, gemini

## Abstract

**Objective:**

Infant growth trajectories, in terms of size, tempo and velocity, may programme lifelong obesity risk. Timing of breastfeeding cessation and weaning are both implicated in rapid infant growth; we examined the association of both simultaneously with a range of growth parameters.

**Design:**

Longitudinal population-based twin birth cohort.

**Subjects:**

The Gemini cohort provided data on 4680 UK infants with a median of 10 (interquartile range=8–15) weight measurements between birth and a median of 6.5 months. Age at breastfeeding cessation and weaning were reported by parents at mean age 8.2 months (s.d.=2.2, range=4–20). Growth trajectories were modelled using SuperImposition by Translation And Rotation (SITAR) to generate three descriptors of individual growth relative to the average trajectory: size (grams), tempo (weeks, indicating the timing of the peak growth rate) and velocity (% difference from average, reflecting mean growth rate). Complex-samples general linear models adjusting for family clustering and confounders examined associations between infant feeding and SITAR parameters.

**Results:**

Longer breastfeeding (>4 months vs never) was independently associated with lower growth velocity by 6.8% (s.e.=1.3%) and delayed growth tempo by 1.0 (s.e.=0.2 weeks), but not with smaller size. Later weaning (⩾6 months vs <4 months) was independently associated with lower growth velocity by 4.9% (s.e.=1.1%) and smaller size by 102 g (s.e.=25 g).

**Conclusions:**

Infants breastfed for longer grew slower for longer after birth (later peak growth rate) but were no different in size, while infants weaned later grew slower overall and were smaller but the timing of peak growth did not differ. Slower trajectories with a delayed peak in growth may have beneficial implications for programming later obesity risk. Replication in cohorts with longer follow-up, alternative confounding structures or randomised controlled trials are required to confirm the long-term effects and directionality, and to rule out residual confounding.

## Introduction

Faster infant growth is an established risk factor for later obesity.^1^ Twin analyses indicate less genetic influence on infancy weight gain than later in childhood, which points towards a critical period when growth is more easily modified by environmental factors.^2,3^ Infant feeding practices are often targeted as modifiable environmental factors for obesity prevention.^4, 5, 6^ However, although systematic reviews of prospective cohorts support a small protective effect of breastfeeding on later obesity,^7,8^ most studies of the age at weaning and subsequent obesity have reported no evidence of association.^9^ Mixed findings from cohort studies are supported by results from randomised controlled trials (RCTs) like PROBIT, a large long-term RCT of breastfeeding exclusivity and duration, which found no differences in obesity prevalence at age 6.5 or 11.5 years.^10,11^ Three shorter-term RCTs of weaning at 6 vs 4 months found no differences in anthropometric outcomes between 6 and 12 months.^12, 13, 14^ There is a suggestion from two US and Danish Cohorts that early weaning (before 4 months) is only associated with later weight gain if breastfeeding also ceased by 4 months,^15,16^ indicating that it may be important to account for both feeding practices concurrently.

Furthermore, single measures of obesity or weight gain between just two time points may not be sufficient to capture the complexity of differences in growth trajectories exhibited by infants in relation to later health risk. For example, it has been reported that breastfeeding is associated with slower growth between 3 and 6 months but faster growth later on,^17,18^ and emerging evidence suggests that the timing of rapid growth (tempo) itself may have an independent role in modifying the risk of later disease.^19^ Similar indicators of developmental timing like adiposity rebound, puberty onset and peak height velocity are associated with a higher risk of adult disease,^20, 21, 22, 23^ suggesting that an accelerated tempo of infant growth may be detrimental to obesity risk.

SuperImposition by Translation And Rotation (SITAR) is a novel method of modelling growth in terms of the size, velocity and tempo of infant weight trajectories.^24^ Two existing studies that have analysed associations between infant feeding and SITAR have reported conflicting results. In 5949 infants from Hong Kong,^25^ longer breastfeeding was associated with a 3% increased growth velocity from birth to 1 year. Conversely, in 602 Australian infants, longer breastfeeding was associated with smaller size and reduced growth velocity from birth to 1 year.^26^ Although infant growth in a rapidly developing economy like Hong Kong, with a different social gradient in infant feeding practices, may genuinely differ from developed economies like Australia, the less detailed measurement of infant feeding in the Hong Kong study may also impact the findings. Neither of these studies has reported the effects of infant feeding on growth tempo or accounted for age at weaning in the same analyses. Furthermore, we are not aware of any investigation of associations between weaning and SITAR parameters, which may shed new light on the role of infant feeding, growth and risk of obesity. Therefore, we aimed to explore the independent associations between infant feeding and the size, tempo and velocity of growth trajectories modelled with SITAR in a large UK twin birth cohort.

## Materials and methods

Data came from Gemini,^27^ a twin birth cohort initiated in 2007 based in England and Wales involving 2402 families (4804 infants) who returned a baseline questionnaire (70% of those contacted and 36% of all eligible families). Parents provided informed written consent and ethical approval was granted by the University College London Committee for the Ethics of non-National Health Service Human Research. All aspects of the data collection and storage were in accordance with the standards stipulated by this body.

Infant feeding practices were assessed in questionnaires completed by the parents at baseline and first follow-up, at mean child ages of, respectively, 8.2 months (s.d.=2.2, range=4–20) and 15.8 months (s.d.=1.1, range=14–27). Duration of breastfeeding (weeks) was calculated as age at cessation minus age at initiation. Three broad levels (none, birth to 4 months, >4 months) and seven detailed levels (none, birth to 1 week, 1 week to 1 month, >1–2 months, >2–3 months, >3–4 months, >4 months) were defined. Age at weaning (first introduction of solid food) was categorised into three broad levels (birth to 4 months, 5 months, 6+ months) and five detailed levels (birth to 3 months, 4 months, 5 months, 6 months, 7+ months).

Infants in England and Wales are measured regularly by health professionals, and weights are recorded in a personal child health record. Gemini parents copied the weights and dates into the questionnaires. A median of 10 (interquartile range (IQR)=8–15) weight measurements per child were reported from birth to median age 6.6 (IQR=5.2–8.3) months. Weights at particular ages: birth (*n*=4639), 3 months (*n*=4214) and 6 months (*n*=3424) were identified for descriptive purposes as those measured closest to, but within 1 month of, that particular age (exact age was recorded). Weight standard deviation scores were calculated adjusted for age, sex and gestational age based on the British 1990 growth reference.^28,29^

**Figure 1:**
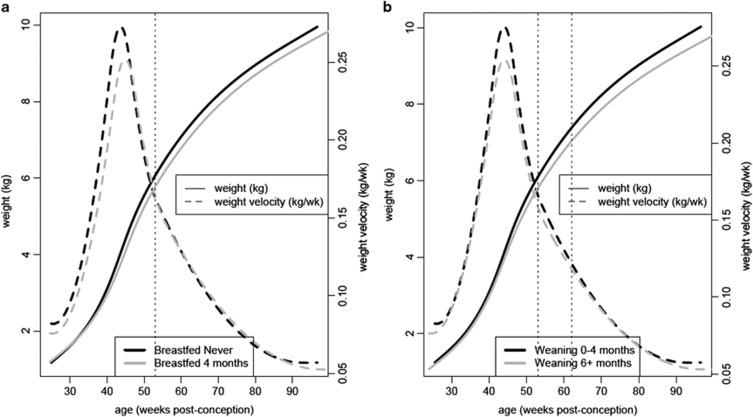
Average weight and weight gain velocity curves for extreme groups of (**a**) breastfed and (**b**) weaned infants. Mean size, tempo and velocity of each group ((**a**) never (black) or >4 months (grey) breastfed; (**b**) 0–4 months (black) or 6+ months (grey) at weaning) were used to plot the average weight (solid lines) and weight gain velocity (dashed lines) curves. Dotted vertical lines represent post-menstrual age in weeks equivalent to categories of breastfeeding duration and age at weaning (that is, age 4 months since birth is 17 weeks + 36 weeks mean gestation=53 weeks post-menstruation).

Weight trajectories were analysed using the SITAR method ^24^ as detailed elsewhere,^3^ the model including all infants who had at least one weight (124 infants had no weight recorded). Briefly, SITAR is a shape invariant model with random effects^26^ that estimates an average growth curve for the sample, plus a set of three parameters for each individual that together transform the average growth curve to match each individual’s growth (for examples of growth curves see Figure 1). Size is expressed in grams with higher values representing larger mean size than average (an upward translation of the weight curve); tempo, the age at peak weight velocity, is expressed in weeks with higher values representing delayed tempo compared with the average tempo (a rightward shift or translation of the weight velocity curve); velocity is expressed as a percentage deviation from mean velocity^30^ with higher values representing faster growth than average (an anticlockwise rotation of the weight curve and an upward translation of the weight velocity curve). The random effects have mean zero and standard deviations estimated from the data. The SITAR analysis was done using a dedicated library written by TJC and based on the nlme library^31^ in the statistical package R,^32^ and the model included fixed effects adjusting for gestation and sex.

Sex, birth order, gestational age, maternal smoking status during pregnancy, maternal education and parental occupation, and dates of birth for children and mothers were reported in the baseline questionnaire. A validated questionnaire^33^ established zygosity. Mothers reported their height and weight at baseline from which body mass index (BMI) was calculated. Socioeconomic status (SES) was indicated by high, medium and low categories of maternal education and parental occupation classified using the National Statistics Socioeconomic Class index.^34^ Parity was represented by the number of other children living with the twins sharing a biological mother.

Analyses were performed in SPSS v17 (SPSS Inc, Chicago, IL, USA). Descriptive analyses are presented for all subjects with data available on infant feeding, using mean and standard deviation (s.d.; continuous and normal), median and IQR (continuous and skewed), or frequencies and percentages (categorical). Complex-samples general linear model assessed associations between growth and infant feeding practices adjusting for family clustering and confounders. Separate models were specified for size, tempo or velocity as the outcome, with independent variables included in a staged approach: (1) either breastfeeding or weaning only, (2) adding twin birth order, sex, zygosity, gestational age, age at baseline (child and mother), parental occupation, maternal education, parity, pregnancy smoking and maternal BMI and (3) adding the other feeding behaviour (that is, breastfeeding and weaning), to identify independent effects. Potential confounders were included if they were associated with both infant feeding and SITAR variables. The reference groups were ‘never breastfed’ and ‘weaned ⩽4 months’.

Linearity of infant feeding associations across categories was explored by replacing each category value with the ‘event age’ (the median age for each weaning or breastfeeding cessation group), and then testing for a linear trend in event age. For this comparison, the detailed breastfeeding and weaning categories were used and regression coefficients represented effects on size, tempo or velocity per week of breastfeeding or month of weaning. Shape plots of size, tempo or velocity vs event age were obtained for breastfeeding and weaning by separately plotting the category means for each growth parameter, adjusted via model 3, against the corresponding event ages. All models were for 4251 infants with complete data on infant feeding, covariates and growth parameters (Supplementary Figure 1).

## Results

Almost a quarter of infants (*n*=1097) were never breastfed; of those breastfed (*n*=3675), 32% were breastfed for more than 4 months (Table 1) but the median duration of breastfeeding was 8 (IQR=4–23) weeks. Exclusive breastfeeding for 1 day or more occurred in 59% of breastfed infants, but the median duration of exclusive breastfeeding was just 4 (IQR=2–21) days. Only 111 infants were exclusively breastfed for more than 4 months (3% of those breastfed, 2% overall). Subsequent analyses are based on exclusive and partial breastfeeding combined.

Age of weaning was 4 months or earlier in 37% and 6 months or later in 28% of the sample (Table 2). Mean age at weaning was 4.6 (s.d.=1.0) months among infants never breastfed, 4.9 (s.d.=1.1) months among those breastfed to 4 months and 5.2 (s.d.=1.0) for those breastfed for more than 4 months (*P*<0.0001). Median age at breastfeeding cessation was 6 (IQR=3–12) weeks among infants weaned between 0 and 4 months, 8 (IQR=3–16) weeks among those weaned at 5 months and 12 (IQR=6–28) weeks for those weaned at 6 months or later (*P*<0.0001). Shorter breastfeeding was associated with being male, monozygotic, younger at baseline and having older siblings, a shorter gestation, and a mother of lower SES, younger, with a higher BMI and smoking in pregnancy (all *P*<0.05, Table 1). Similar associations were observed for earlier weaning except that zygosity was unrelated, and longer gestation was associated with earlier weaning (Table 2).

Earlier weaning was associated with a higher weight standard deviation score at birth, 3 and 6 months (Table 2), whereas shorter breastfeeding was associated with a higher weight standard deviation score at 3 and 6 months only (Table 1). Figure 1 illustrates SITAR model-1-based mean weight and weight velocity curves for extreme groups of breastfed and weaned infants, illustrating the clear peak in velocity soon after 40 post-menstrual weeks. The groups were similar at birth, but clear differences emerged by 90 post-menstrual weeks (approximately 12 months since birth), suggesting that both never breastfeeding and earlier weaning (solid black lines) are associated with larger size compared with breastfeeding for 4 months or more and weaning at 6 months or later. For never breastfed infants, the age at peak weight velocity is earlier (Figure 1a, dashed black line), signifying an advanced growth tempo compared with infants breastfed for longer (Figure 1a, dashed grey line). Both never breastfed infants and those weaned earlier (black dashed lines) displayed higher peaks in weight velocity indicating a faster growth trajectory than infants breastfed for longer or weaned later (dashed grey lines).

**Figure 2:**
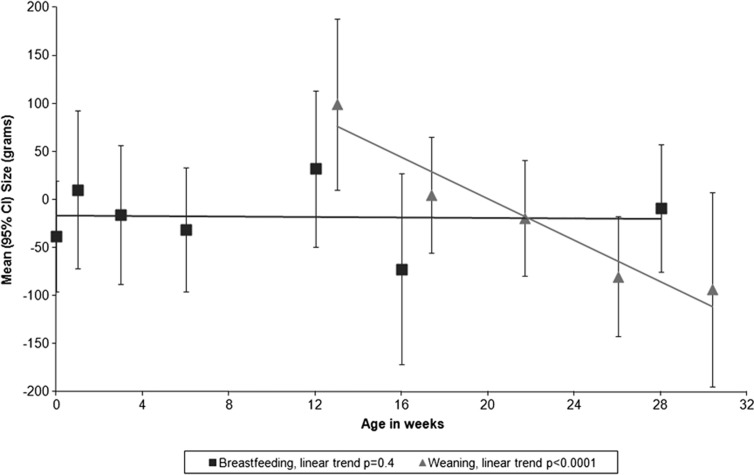
Size by age at weaning or cessation of breastfeeding group mean (95% confidence interval (CI)) plotted against the group median event age (age at weaning or cessation of breastfeeding). Means derived from complex-samples general linear model with size as the outcome; independent variables median age at cessation of breastfeeding (seven groups: (i) 0 weeks; (ii) 1 week; (iii) 3 weeks; (iv) 6 weeks; (v)12 weeks; (vi) 16 weeks; (vii) 28 weeks) and median age at weaning (five groups: (i) 3 months; (ii) 4 months; (iii) 5 months; (iv) 6 months; (v) 7months) adjusting for clustering of twins within families and twin order, sex, zygosity, gestational age, age at baseline (child and mother), parental occupation, maternal education, parity, pregnancy smoking, BMI. Tests for linear trend were performed by comparing models with the feeding practice variable categories coded either as median event age or level.

In adjusted models with the SITAR parameter size as the outcome, later weaning, but not longer breastfeeding, was independently associated with smaller size; infants weaned at or after 6 months were 102 g (s.e.=25 g) smaller than those weaned by 4 months (Supplementary table 1, model 3). The relationship was linear, with size decreasing by 46 g (s.e.=11 g) for each extra month that weaning was postponed (Figure 2).

**Figure 3:**
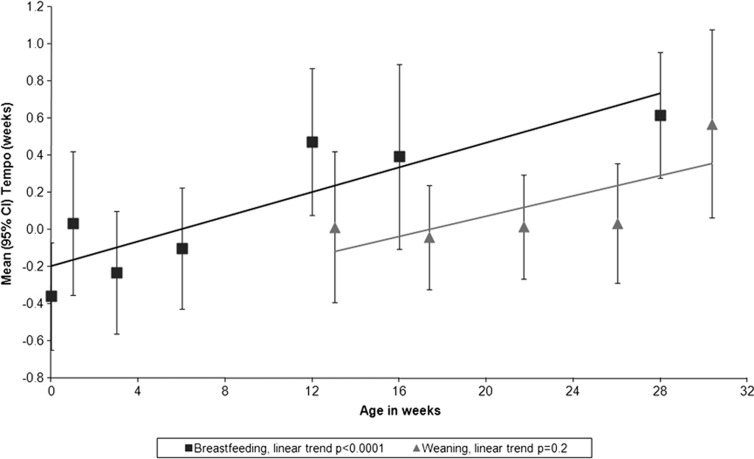
Tempo by age at weaning or cessation of breastfeeding. Group mean (95% confidence interval (CI)) plotted against the group median event age (age at weaning or cessation of breastfeeding). Means derived from complex-samples general linear model with tempo as the outcome; independent variables median age at cessation of breastfeeding (seven groups: (i) 0 weeks; (ii)1 week; (iii) 3 weeks; (iv) 6 weeks; (v)12 weeks; (vi) 16 weeks; (vii) 28 weeks) and median age at weaning (five groups: (i) 3 months; (ii) 4 months; (iii) 5 months; (iv) 6 months; (v) 7months) adjusting for clustering of twins within families and twin order, sex, zygosity, gestational age, age at baseline (child and mother), parental occupation, maternal education, parity, pregnancy smoking, BMI. Tests for linear trend were performed by comparing models with the feeding practice variable categories coded either as median event age or level.

In adjusted models with the SITAR parameter tempo as the outcome, longer breastfeeding was independently associated with delayed growth tempo; growth rates of infants breastfed for more than 4 months peaked 1.0 (s.e.=0.2 weeks) later than those never breastfed (Supplementary Table1, model 3). The relationship was linear, with tempo delayed by 0.03 (s.e.=0.01 weeks) per extra week of breastfeeding (Figure 3). A weak association between age at weaning and tempo in the unadjusted model (infants weaned at or after 6 months peaked 0.3 (s.e.=0.1) weeks later than those weaned by 4 months, *P*=0.04) was attenuated after the inclusion of covariates (Supplementary Table 1, models 2 and 3) and there was no linear trend (Figure 3).

**Figure 4:**
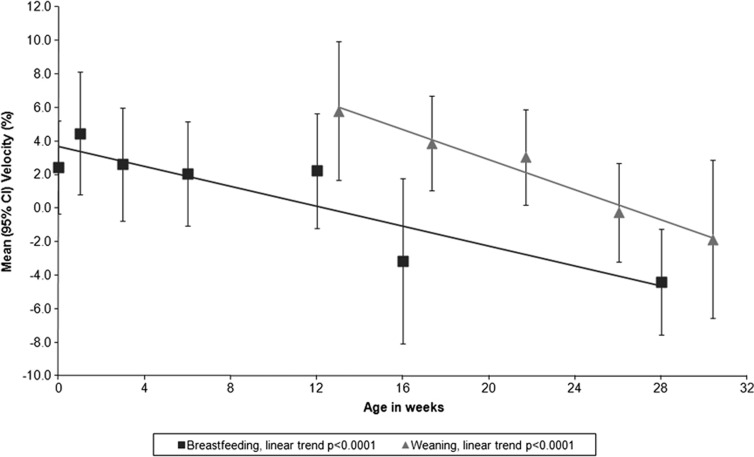
Velocity by age at weaning or cessation of breastfeeding. Group mean (95% confidence interval (CI)) plotted against the group median event age (age at weaning or cessation of breastfeeding). Means derived from complex-samples general linear model with velocity as the outcome; independent variables median age at cessation of breastfeeding (seven groups: (i) 0 weeks; (ii) 1 week; (iii) 3 weeks; (iv) 6 weeks; (v) 12 weeks; (vi) 16 weeks; (vii) 28 weeks) and median age at weaning (five groups: (i) 3 months; (ii) 4 months; (iii) 5 months; (iv) 6 months; (v) 7months) adjusting for clustering of twins within families and twin order, sex, zygosity, gestational age, age at baseline (child and mother), parental occupation, maternal education, parity, pregnancy smoking, BMI. Tests for linear trend were performed by comparing models with the feeding practice variable categories coded either as median event age or level.

In adjusted models with the SITAR parameter velocity as the outcome, both later weaning and longer breastfeeding were independently associated with lower growth velocity; infants weaned at or after 6 months grew 4.9% (s.e.=1.1%) slower than infants weaned by 4 months, and infants breastfed for more than 4 months grew 6.8% (s.e.=1.3%) slower than those never breastfed (Supplementary Table 1, model 3). Both relationships were linear (Figure 4), with growth velocity 2.2% (s.e.=0.5%) slower per extra month that weaning was postponed and 0.3% (s.e.=0.04%) slower per extra week of breastfeeding.

Sensitivity analyses were performed to check if the results were altered by mutual adjustment for SITAR parameters, or by the reduction in sample size caused by the inclusion of covariates, but the pattern of results remained the same (Supplementary Table 1, models 4 and 5). All models were repeated in a restricted sample of twins born at term (gestational age ⩾37 weeks) and the pattern of results was unaltered (data not shown).

## Discussion

In this study of infant feeding and growth trajectories, longer breastfeeding and later weaning were both associated with lower growth velocity, and additionally with delayed tempo and smaller size, respectively.

Growth velocity was 6.8% greater in those never breastfed vs those breastfed for more than 4 months, which is a slightly smaller effect than observed in an Australian study^26^ and contrasts with results from the Hong Kong cohort that found slower growth among less breastfed infants.^25^ Both of those studies modelled growth using SITAR, but the Hong Kong study characterised breastfeeding using a single cutoff of 1 month for exclusive breastfeeding, which may have lacked sensitivity. In Gemini and the Australian study, breastfeeding duration was recorded in more detail and specifically separated infants never breastfed from those breastfed for any duration. The SES patterning of weight/feeding differs in Hong Kong such that differences in associations might be explained by residual confounding by social class, which cannot be completely adjusted for in Gemini.^35^ However, infant feeding could plausibly be a mediator rather than a confounder of the relationship between SES and growth. We have demonstrated in earlier analyses of Gemini that the SES effect on weight change from birth to 3 months is attenuated primarily by adjustment for breast feeding rather than other SES-related factors like maternal smoking in pregnancy or parental BMI.^36^

In previous studies using SITAR, the effect of feeding on tempo was not reported (potentially because the importance of the timing of changes in growth was not recognised at that time) making it impossible to directly compare our results. Growth tempo, which is effectively the age when infant weight velocity peaks, is similar to subsequent developmental indicators such as the timing of adiposity rebound or the onset of puberty, which have evidence of an important role in programming later obesity and chronic disease risk.^19, 20, 21, 22, 23^ Previous research has shown that prolonged breastfeeding in Filipino girls was associated with delayed age at menarche, with the likelihood of early menarche falling by 6% per extra month of exclusive breastfeeding.^37^ In addition, breastfeeding for more than 4 months was associated with a 10-month delay in the age at adiposity rebound in children from an Australian cohort.^38^ These associations of longer breastfeeding with delayed development match results from Gemini and suggest that infant growth tempo could be an early indicator of developmental timing.

Infant feeding and growth were both measured longitudinally, but because they were modelled concurrently the direction of effect cannot be inferred, which is a limitation of our study. Feeding and growth are likely to be related in both directions, with transitions to formula or solid food potentially occurring in response to earlier growth rates^39^ as well as impacting on subsequent growth.^18^ Mothers may struggle to maintain breastfeeding when the infant’s rate of weight gain (and energy requirement and demand for food) is near its peak. Therefore, an advanced tempo might encourage earlier breastfeeding cessation because mothers conclude that breast milk alone is not enough to satisfy her infant’s appetite. Larger size and birth weight were related to earlier weaning, which raises the possibility of reverse causation in the weaning–growth association, that is, that babies who are born large grow faster and tend to be weaned earlier, because the larger size at birth precedes the exposure to early weaning. In contrast, breastfeeding initiation or duration is not associated with birth weight, indicating that the breastfeeding exposure is non-differential by size and occurs before the observed delay in tempo and slower growth trajectory. However, the causal nature of these associations should be explored using alternative study designs such as randomised controlled trials.

Although many potential confounders were controlled for, residual confounding remains a possibility. Sib-pair analyses are a good strategy to reduce the residual confounding effects of common maternal factors, for example, SES,^40^ but discordant feeding practice within twin pairs was rare in Gemini, and the informative sample size was insufficient to detect within-family associations. Arguably, within-pair differences in feeding may reflect individual infant factors, such as poor appetite or illness, or other problems related to growth that precede feeding method choices, which may limit the ability of a sib-pair design to completely address such queries. Ultimately, experimental studies involving randomisation of breastfeeding duration or weaning age, such as PROBIT^10^ and a recent Icelandic study,^14^ are needed to clarify remaining questions around the nature of causality in the associations observed in the current study.

The main limitation of the study is the use of a twin sample. Twins tend to be born smaller than singletons and subsequently grow faster.^41^ However, within twins the environmental causes of differences in growth should be similar, and associations between growth and feeding practices have not been noted to be different among twins vs singletons. Long-term health does not differ among twins compared with singletons, suggesting that the systematic difference in growth does not lead to fundamentally different health risks^42^ and that variation in growth rates within a twin sample are subject to the same causes and consequences as in singletons. The demands of breastfeeding are greater for mothers of twins, which may result in earlier cessation, but similar levels of variation in breastfeeding were observed in Gemini as in a national survey. The proportion of ever breastfed infants in Gemini (born in 2007) was 77% compared with 69% and 81% for multiple and singleton infants, respectively, in the UK 2010 Infant Feeding Survey.^43^ It was not possible to analyse associations with exclusive breastfeeding, because only 2% of Gemini infants were exclusively breastfed beyond 4 months, which restricted the power for estimating differences in growth trajectories. This issue is not limited to twin studies, however, as a comparable proportion of UK singletons (3–8%) are exclusively breastfed for 4 months.^44^ Another drawback, which exists in other cohort studies,^45,46^ is that higher SES families are over-represented in Gemini, thus the representative nature of the findings is limited to similar populations. This limitation is compounded by missing covariate data in 12% of the baseline sample, which excluded twins from lower SES families from the final analysis and with a shorter gestation (Supplementary Tables 2 and 3). Given that SES differences in infant feeding and weight gain have been observed in this sample,^36^ selection bias may have caused the associations between infant feeding and growth to be underestimated. However, unadjusted associations in those with available data on infant feeding and growth (*n*=4680) were similar to those in the sample with complete data (models 1 and 5, Supplementary Table 1).

The study is strengthened by having multiple weight measures, which increases the accuracy of growth trajectory estimates over measures from just one or two time points. SITAR accounts for the nonlinear shape of infant growth and has the advantage of efficiently estimating growth trajectories and using all the available data irrespective of measurement timing or frequency. Weight was measured by health professionals and extracted from child records, a process that compares well with clinic-based measures.^47^ The collection of infant feeding data during infancy is a strength because the potential for recall bias was reduced compared with studies that collected data after infancy.^48^ Maternal reporting of breastfeeding can be affected by social desirability and many other influences, which may result in both under- and over-reporting of breastfeeding duration.^48^ Random error reduces the study’s power to detect effects of a given size, suggesting that our results may underestimate the true association between infant feeding and growth. Finally, the application of SITAR to infant growth data is still relatively new. It is unclear whether the growth trajectory associated with longer breastfeeding and later weaning is optimal in terms of later health, although previous work characterising similar concepts like rapid infant growth and timing of adiposity rebound or puberty^19, 20, 21, 22, 23^ suggest that lower velocity and delayed tempo of growth would be beneficial to health. Future work should investigate how SITAR parameters relate to growth, body composition and health beyond infancy.

## Conclusion

Longer breastfeeding and later weaning were both associated with lower growth velocity. Longer breastfeeding was also associated with delayed tempo, and later weaning with smaller size. These results support the hypothesis that longer breastfeeding and later weaning are associated with slower growth, but further research is needed to establish whether these associations are causal and if they could be modified to lower subsequent obesity risk.

## Figures and Tables

**Table 1:** Summary statistics of growth and covariates by duration of breastfeeding in Gemini

	*Category*	*Breastfeeding duration (max,* n*=4772)*								
		*Never* n*=1097 (23%)*			*Birth to 4 months* n*=2507 (53%)*			*>4 months* n*=1168 (24%)*		
		N	n	*%*	N	n	*%*	N	n	*%*
Sex	Female^a^	1097	513	47	2507	1255	50	1168	632	54
Zygosity	Monozygotic^b^	1071	361	34	2431	766	32	1136	325	29
Twin birth order	1st Born	1097	543	50	1258	689	55	1168	585	50
Parity	No older siblings^a^	1067	378	35	2430	1444	59	1135	556	49
Maternal age	<30 Years old^a^	1095	395	36	2499	681	27	1166	176	15
Maternal smoking in pregnancy	Ever smoked^a^	1095	227	21	2507	265	11	1166	44	4
Socioeconomic status	High occupation^a^	1089	435	40	2503	1691	68	1164	894	77
	High education^a^	1097	168	15	2507	1080	43	1168	752	64
Weaning	6+ Months^a^	1088	220	20	2483	675	27	1162	459	40
		N	*Mean*	*s.d.*	N	*Mean*	*s.d.*	N	*Mean*	*s.d.*
Maternal BMI (kg m^−^^2^)^a,d^		1060	26	5.4	2446	25.4	4.8	1140	24.1	4.1
Age at baseline (months)^a,c^		1097	8	2.1	2507	8.1	2.2	1168	8.4	2.2
Gestational age (weeks)^a,d^		1093	36.3	2.1	2496	35.9	2.7	1163	36.7	2.4
Weight SDS	Birth^c^	1060	−0.53	0.96	2429	−0.56	0.96	1148	−0.58	0.92
	3 Months^a^^c^	899	−0.09	1.04	2242	−0.22	1.09	1071	−0.56	1.09
	6 Months^a^^c^	710	−0.1	1.09	1816	−0.17	1.12	898	−0.55	1.1
SITAR	Size (grams)^d^	1069	−10	525	2454	6	486	1157	−4	563
	Tempo (weeks)^a^^d^	1069	−0.4	2.6	2454	−0.1	2.4	1157	0.5	3.1
	Velocity (%)^a^^d^	1069	2.2	21.3	2454	1.6	21.2	1157	−5.4	22.8

Abbreviations: BMI, body mass index; SDS, standard deviation score; SITAR, SuperImposition by Translation And Rotation.

*N* is the sample size with data available. *n* is the sample size in the specified category. % is proportion in the specified category out of the total sample with data available, that is, (*n/N*)*100.

a Differences across groups were significant at *P*<0.0001.

b Differences across groups were significant at *P*<0.05. Differences across breastfeeding categories were tested by

c one-way analysis of variance or

d Kruskal–Wallis test for continuous variables, and by *χ*^2^ test for categorical variables.

**Table 2:** Summary statistics of growth and covariates by age at weaning in Gemini

	*Category*	*Age at weaning (max,* n*=4745)*								
		*Birth to 4 months,* n*=1714 (36%)*			*5 months,* n*=1667 (35%)*			*6+ months,* n*=1364 (29%)*		
		N	n	*%*	N	n	*%*	N	n	*%*
Sex	Female^a^	1714	772	45	1667	852	51	1364	759	56
Zygosity	Monozygotic	1663	542	33	1620	484	30	1330	424	32
Twin order	1st born	1714	861	50	1667	840	50	1364	671	49
Parity	No older siblings^b^	1678	790	47	1601	886	55	1328	690	52
Maternal age	<30 Years old^a^	1710	616	36	1663	392	24	1360	236	17
Maternal smoking in pregnancy	Ever smoked^a^	1714	271	16	1665	142	9	1362	116	9
Socioeconomic status	High occupation^a^	1710	917	54	1657	1154	70	1362	935	69
	High education^a^	1714	518	30	1667	790	47	1364	682	50
Breastfeeding	>4 months^a^	1714	256	15	1665	447	27	1354	459	34
		N	*Mean*	*s.d.*	N	*Mean*	*s.d.*	N	*Mean*	*s.d.*
Maternal BMI (kg/m2)^a,d^		1658	26	5.3	1633	24.7	4.5	1332	24.4	4.4
Age at baseline (months)^a,d^		1714	8	2.2	1667	8	2.1	1364	8.6	2.3
Gestational age (months)^a,d^		1704	36.6	2.2	1663	36.3	2.4	1358	35.7	2.8
Weight SDS	Birth^a^^c^	1669	−0.49	0.95	1617	−0.58	0.94	1312	−0.61	0.94
	3 Months^a^^c^	1485	−0.1	1.08	1514	−0.28	1.08	1180	−0.49	1.09
	6 Months^a^^c^	1120	−0.07	1.13	1267	−0.25	1.06	1021	−0.46	1.11
SITAR	Size^a^^c^	1682	42	538	1633	2	513	1326	−52	480
	Tempo^b^^c^	1682	−0.2	2.56	1633	0.1	2.62	1326	0.1	2.7
	Velocity^a^^d^	1682	2.8	22.5	1633	0.1	20.9	1326	−3.4	21.1

Abbreviations: BMI, body mass index; SDS, standard deviation score; SITAR, SuperImposition by Translation And Rotation.

*N* is the sample size with data available. *n* is the sample size in the specified category. % is proportion in the specified category out of the total sample with data available, that is, (*n/N*)*100.

a Differences across groups were significant at *P*<0.0001.

b Differences across groups were significant at *P*<0.05. Differences across breastfeeding categories were tested by

c one-way analysis of variance or

d Kruskal-Wallis test for continuous variables, and by *χ*^2^ test for categorical variables.
