# Supplementary material for: Associations between infant feeding and the size, tempo and velocity of infant weight gain: SITAR analysis of the Gemini twin birth cohort
Source: Int J Obes (Lond). 2014 May 6;38(7):980–7. doi: 10.1038/ijo.2014.61 (PMC4088337; doi:10.1038/ijo.2014.61)
Supplement: Supplementary Information [file ijo201461x1.doc]

**File contents:**

Figure 1: Flow chart describing data available for analysis.

Table 1: Results from complex-samples general linear modelling of infant feeding practice categories in relation to SITAR parameters (size, tempo, velocity).

Table 2: Descriptive statistics among those with any baseline data vs. those with complete data on growth, infant feeding and covariates.

Table 3: Multivariable logistic regression of being included in the analysis sample.

Figure 1: Flow chart describing data available for analysis.

Full cohort of twins recruited

n = 4804

Breastfeeding

n = 4772

Growth (SITAR)

n = 4680

Weaning

n = 4745

Breastfeeding, Weaning, Growth

n = 4641

Breastfeeding, Weaning, Growth

& potential confounders

n = 4251

Breastfeeding and Growth

n = 4680

Weaning and growth

n = 4641

**Supplementary table 1:** Associations between infant feeding practices and SITAR size, tempo and velocity

*Regression coefficients (RC) and standard errors (SE) from a complex samples general linear model with each SITAR parameter as the outcome and breastfeeding or weaning as the exposure, taking into account clustering of twins within families. Model 1 either breastfeeding or weaning category only; Model 2 is model 1 plus twin order, sex, zygosity, gestational age, age at baseline (child and mother), parental occupation, maternal education, parity, pregnancy smoking and BMI; Model 3 is model 2 but includes both breastfeeding and weaning categories. Model 4 is model 3 but includes the non-outcome SITAR parameters as covariates. Model 5 is Model 1 in the sample all available data on infant feeding, growth but not potential confounders. Reference groups were ‘never breastfed’ and ‘weaned at or before 4 months’.*

**Supplementary table 2:** Descriptive statistics among those with any baseline data vs. those with complete data on growth, infant feeding and covariates

**Supplementary table 3:** Multivariable logistic regression of being included in the analysis sample (n=4650).
